# Lifestyle and risk factors associated with elevated prostate-specific antigen levels in rural men: implications for health counseling

**DOI:** 10.3389/fonc.2024.1451941

**Published:** 2024-09-23

**Authors:** Kun-Lu Hsieh, Chia-Hao Chang, Yu-Chih Lin, Tung-Jung Huang, Mei-Yen Chen

**Affiliations:** ^1^ Department of Family Medicine, Chang Gung Memorial Hospital, Chiayi, Taiwan; ^2^ Department of Nursing, Chang Gung University of Science and Technology, Chiayi, Taiwan; ^3^ Department of Pulmonary and Critical Care, Chang Gung Memorial Hospital, Yunlin, Taiwan; ^4^ Research Fellow, Department of Cardiology, Chang Gung Memorial Hospital, Chiayi, Taiwan; ^5^ School of Nursing, Chang Gung University, Taoyuan, Taiwan

**Keywords:** prostate-specific antigen, prostate cancer, propensity score matching, genetic matching, cardiometabolic disease, waist-hip ratio, water intake

## Abstract

**Background:**

The use of prostate-specific antigen (PSA) for early detection of prostate cancer (PCa) is common but controversial. In rural areas, PSA is widely used for screening because it is convenient and early-stage PCa often shows no symptoms. Studies suggest that PSA levels are linked to factors like unhealthy lifestyles, obesity, lack of exercise, inflammation, and aging. Proper use and interpretation of PSA are crucial for healthcare providers, especially in primary care settings. This study aims to explore the prevalence and factors linked to higher PSA levels in rural men.

**Methods:**

We conducted a community-based cross-sectional study from March to December 2023 in the western coastal region of Taiwan. Men aged 40-75 years participated, completing a lifestyle questionnaire and providing blood samples for cardiometabolic biomarkers and PSA levels. PSA levels of ≥ 4.0 ng/mL were considered elevated. We used propensity score matching (PSM) and genetic matching (GM) for analysis, followed by regression analysis.

**Results:**

In total, 3347 male adults with a mean age of 56.3 years (SD=11.8, range 40-75), and without cancer-related diseases, were enrolled. Findings indicated that 3.9% (n=130) of men aged 40-75 years had a PSA ≥ 4 ng/mL. and many of them did not adopt health-related behaviors, including inadequate servings of vegetables, water intake, and engaging in regular exercise. Furthermore, more than half of the participants had high blood pressure, and over one-quarter exhibited a higher waist-hip ratio and cardiometabolic diseases. After employing propensity score matching (PSM) and genetic matching (GM) with respect to age and education, the multivariate logistic regression model indicated that less water intake (p<0.01), higher waist-hip ratio (> 0.95) (p<0.05), and being diagnosed with cardiometabolic diseases (p<0.05) were significantly associated with a higher serum PSA level.

**Conclusion:**

This study revealed that inadequate water intake and obesity related diseases are significant risk factors associated with elevated PSA levels among male adults living in rural areas. It is important for frontline healthcare providers to carefully interpret the meaning of a high PSA level. Additionally, launching a longitudinal study is necessary to further investigate its relation to PCa.

## 1 Introduction

Prostate cancer (PCa) is the second most common malignancy worldwide. Approximately 1.4 million new cases of PCa are documented annually. In 2022, the global standardized incidence rate was 29.4 and accounted for an age-standardized mortality of 7.3 per 10^5^ men. PCa is the fifth leading cause of cancer-related mortality among men worldwide (1–4). In Taiwan, PCa is the fifth leading cause of cancer-related deaths in men, and the standardized incidence and mortality rates have been reported to be 35.3 and 7.4 per 10^5^ men, respectively. Notably, both the incidence and mortality rates of PCa have shown an increasing trend annually (5, 6).

The World Health Organization has announced that nearly 50% of cancers can be prevented by adopting healthy lifestyle choices. These preventive measures include abstaining from tobacco and alcohol, engaging in regular physical activity, and maintaining a healthy weight, as evidenced by metrics such as waist circumference and waist-to-hip ratio (WHR) (7). Literature indicates that smoking, obesity, high lipid levels, and significant dairy consumption are associated with an increased risk of advanced PCa. Conversely, physical activity has been shown to significantly reduce this risk (8–10). Additionally, increased consumption of a plant-based diet is associated with a reduced risk of aggressive forms of PCa in men aged < 65 years (11).

Prostate-specific antigen (PSA) is the most widely used biomarker for PCa, serving as a common tool for early detection and monitoring of PCa progression (3, 8, 12). Clinically, PSA is used as a screening tool for PCa with a cut-off value of 4 ng/mL. This screening approach has helped reduce the progression to advanced PCa and potentially decreases the risk of death from PCa in men (3, 13–16). The American Urological Association and American Cancer Society guidelines continue to include PSA screening for PCa diagnosis, which recommend yearly screening for men aged over 40 years and whose PSA level is > 2.5 ng/mL (14, 17, 18).

PSA is a kallikrein-like serine protease produced by the epithelial cells of all types of prostatic tissues, including benign and malignant tissues. Its primary role is to liquefy the ejaculate and enhance sperm motility (12, 19, 20). In serum, PSA primarily exists in three forms: complexed with two serine protease inhibitors—α1-antichymotrypsin (PSA-ACT) and α2-macroglobulin (PSA-MG), as well as in a free or unbound state (free PSA). Abnormal elevation of PSA levels is often associated with malignant prostate tumors. Studies have shown that age is significantly associated with PSA levels. As age increases, PSA levels in healthy men tend to rise correspondingly. In addition, other medical factors such as benign prostatic hyperplasia, prostatitis, urinary retention, and the use of medical devices (e.g., urethral catheterization, transurethral ultrasound, and prostate biopsy) can also contribute to elevated PSA levels (15, 20, 21). Conversely, low PSA levels are often associated with the consumption of a healthy plant-based diet and regular physical activity (22, 23). Few studies have explored the risk factors associated with PSA levels in Taiwan. From a health promotion perspective and with regard to frontline healthcare providers, the proper use and interpretation of this tumor marker are crucial issues, especially in the early stages of the disease, since many patients with PCa do not experience symptoms. Hence, the purpose of this study was to explore the significance and risk factors associated with elevated serum PSA levels in rural Taiwanese men.

## 2 Materials and methods

### 2.1 Design and population

This series of studies was designed to match health needs and facilitate the establishment of an evidence-based health promotion program for middle-aged men in rural Taiwan. An annual community health screening was conducted between March and December 2022 in western coastal Yunlin County, Taiwan. Adult community residents were encouraged to participate in collaboration with a local hospital and the health district. The analyzed data included (1) male sex and age of 40-75 years, (2) completed questionnaires, and (3) receipt of participants’ informed consent. Participants diagnosed with cancers such as prostate or gastrointestinal tract cancer were excluded from the analysis.

### 2.2 Procedure and ethical considerations

This study was approved by the Institutional Review Board (IRB) of the Research Ethics Committee (IRB no: 202000109B0C101). Each participant was informed of the purpose and procedures related to the study, such as fasting for at least 8 hours overnight. Face-to-face questionnaire-based interviews were conducted by six registered nurses trained by the primary investigator. Blood samples were collected and stored according to the standard procedures of the central laboratory of the collaborating local hospital.

### 2.3 Measurements

*Demographic characteristics and health histories* included age, level of education (years of education received), height (cm), body weight (kg), waist and hip circumferences (cm), alcohol consumption (yes: regular; no: never or on special days only), cigarette smoking (yes: current/former smoker; no: never), and the most common 11 multi-comorbidities (including cancer, diabetes, hypertension, coronary heart disease, and stroke). Participants who reported hypertension, coronary heart disease, diabetes, hyperlipidemia, or stroke were categorized as having cardiometabolic disease (CMDs).

*Health-related behaviors* included the frequency of (1) at least three servings of vegetables and two servings of fruit in the diet per day. One serving of cooked vegetables is approximately half a bowl, and one serving of fruit is about the size of a fist. (2) intake of at least 2000 mL of plain water per day, (3) performance of regular exercise at least three times and 30 min each time per week with responses categorized as “low: never or seldom” and “high: usually or always.” The recommended intake of vegetables and fruits, water consumption, and exercise frequency in this study were based on the health guidelines provided by the Health Promotion Administration (HPA) of Taiwan (25–27).

*Physiological biomarkers* included the following: (1) WHR, (2) systolic and diastolic blood pressure values, (3) fasting serum high-density lipoprotein cholesterol (HDL-C) levels (mg/dL), and (4) fasting serum triglyceride levels (mg/dL).

*PSA* (ng/mL) was defined as the sum of the levels of PSA complexed with a1-antichymotrypsin (PSA-ACT) and unbound PSA (free PSA). These two forms of PSA were measured by electrochemiluminescence immunoassay on a COBAS E601 analyzer. Based on previous studies, the cut-off value for a high serum PSA level was set at ≥ 4.0 ng/mL (3, 8). Central obesity was defined as a waist-to-hip ratio (WHR) of 0.95 or greater; therefore, we set the cutoff value for WHR at ≥0.95 (7).

### 2.4 Statistical analyses

Continuous variables are presented as mean ± standard deviation values and were compared between groups using independent sample t-tests. Categorical variables are presented as frequencies and percentages and were compared using the chi-square test. Considering the small sample size of abnormal PSA levels, which are markedly influenced by age and demographic factors, two distinct multivariate analysis methods were employed to assess the primary outcomes of this study: traditional covariate-adjusted binary logistic regression analysis of propensity score matching (PSM) and genetic matching (GM). Applying PSM and GM to create comparable data matching should reduce potential confounding and optimizes the balance of covariates between groups.

PSM typically offers more advantages compared with traditional regression analysis by allowing for the calculation of propensity scores. Propensity scores reduce the entire covariate distribution to a single dimension, implying that two units with similar propensity scores may not necessarily have similar covariate values. Nonetheless, PSM typically achieves a good covariate balance (24). Applying this concept to the present study, if units could be identified in the PSA of ≥ 4 ng/mL group that closely align with units in the PSA of < 4 ng/mL group, each pair would have similar covariate values (age and education), and the covariate distribution in these two groups in the matched sample will be similar. By balancing these covariates, the study can more confidently attribute differences in PSA levels to the variables of interest. GM is a sophisticated multivariate matching approach developed by Sekhon and Mebane. It employs optimization techniques to identify the most suitable distance metric for achieving optimal balance. King and Nielsen contended that GM could achieve superior levels of balance and decreased bias compared with PSM. In this study, GM was used to further refine the balance of covariates between the groups. By optimizing the matching process, GM aims to ensure that the groups are as comparable as possible, minimizing residual confounding that might still exist after PSM. This allows for more robust conclusions about the associations between lifestyle factors, CMDs, and elevated PSA levels. The R packages “matchIt,” “matching,” and “cobalt” were used to perform PSM and optimization using the genetic algorithm (28). An alpha level of 0.05 was considered to indicate statistical significance. All statistical analyses were performed using R version 4.3.1 (Vienna, Austria).

## 3 Results

### 3.1 Demographics and characteristics of the participants

Overall, 3347 rural men (mean age: 56.3 ± 11.8 years; range: 40-75 years) were enrolled in this study, and 3.9% (n = 130) were found to have PSA levels of ≥4 ng/mL. **Table 1** shows that 57% of participants received primary school level of education; 46% and 41% did not consume adequate servings of fruits and vegetables, respectively, 27.8% had inadequate water intake, and 41.7% did not regularly exercise. Furthermore, >50% of men had high blood pressure (51.8%), 27.8% had a high WHR, and 29.2% were diagnosed with CMDs. Univariate analysis revealed that the following factors were associated with high PSA levels: old age (*P* <.001), receipt of few years of education (*P* <.001), adoption of a low frequency of vegetable consumption (*P* <.05), low water intake (*P* <.01), high WHR (*P* <.001), and a diagnosis of CMD (*P* <.001). Multivariate logistic regression model analysis showed that old age (*P* <.001) low water intake (*P* <.01), a high WHR (*P* = .05), and a diagnosis of CMD (*P* <.05) significantly affected high serum PSA levels (**Table 2**).

**Table 1:** Demographic characteristics and health-related information among participants.

Variables	PSA<4 (n=3217)^4^ n %	PSA≥4 (n=130) n %	P
Age (years, mean, SD)	47.5 ± 17.7	67.4 ± 11.2	<0.001
Education level			<0.001
Low (primary school)	1729 (54)	99 (77)	
High (≥ middle school )	1350 (46)	30 (23)	
Vegetables (3 servings/day)*			0.04
Low	1307 (42)	66 (51)	
High	1801 (58)	63 (49)	
Fruits (2 servings/day)*			0.66
Low	1499 (48)	60 (47)	
High	1593 (52)	69 (53)	
Water intake (2000 mL/ day)*			0.002
Low	877 (28)	53 (41)	
High	2217 (72)	76 (59)	
Exercise (30 min/3 times/week)*			
Low	1350 (44)	47 (37)	0.14
High	1748 (56)	80 (63)	
Smoke (current/former)			
Yes	1059 (34)	39 (30)	0.36
No	2042 (66)	90 (70)	
Alcohol drinking			
Yes	867 (28)	32 (25)	0.44
No	2236 (72)	97 (75)	
Waist-hip ratio (WHR)			<0.001
≧0.95	866 (27)	65 (50)	
<0.95	2327 (73)	64 (50)	
SBP/DBP (mmHg)^1^			0.09
≧130/85	1658 (52)	77 (59)	
<130/85	1548 (48)	53 (41)	
HDL-C (mg/dL)^2^			0.75
< 40 mg/dL	580 (18)	22 (17)	
≧40 mg/dL	2636 (82)	108 (83)	
TG (mg/dL)^3^			0.63
≧150	854 (27)	37 (28)	
<150	2362 (73)	93 (72)	
Cardiometabolic diseases			<0.001
Yes	900 (29)	76 (59)	
No	2188 (71)	53 (41)	

*Low: never or seldom; High: usually or always.

^1^systolic/diastolic blood pressure; ^2^high-density lipoprotein-cholesterol; ^3^triglyceride; ^4^prostate-specific antigens.

**Table 2:** Chi-squared test and logistic regression analysis before genetic matching (full data).

Variables	Univariate			Multivariate		
	PSA<4 (N=3217) n (%)	PSA≥4 (N=130) n (%)	*p*	effect	SE	*p*
Age (years)			<0.001			<0.001
>50	1786 (56)	121 (93)		2.58	0.37	
≤50	1431 (44)	9 (7)		–	–	
Education level			<0.001			0.51
Low	1729 (54)	99 (77)		0.15	0.23	
High	1350 (46)	30 (23)		–	–	
Vegetables (3 servings/day)			0.04			0.28
Low	1307 (42)	66 (51)		0.21	0.19	
High	1801 (58)	63 (49)		–	–	
Water intake (2000 mL/day)			0.002			0.003
Low	877 (28)	53 (41)		0.57	0.19	
High	2217 (72)	76 (59)		–	–	
Waist-hip ratio (WHR)			<0.001			0.05
>0.95	866 (27)	65 (50)		0.37	0.19	
<0.95	2327 (73)	64 (50)		–	–	
Cardiometabolic diseases			<0.001			0.04
Yes	900 (29)	76 (59)		0.39	0.20	
No	2188 (71)	53 (41)		–	–	

PSA, prostate-specific antigen.

### 3.2 Analysis of PSA levels using propensity score matching and logistic regression

**Figure 1:**
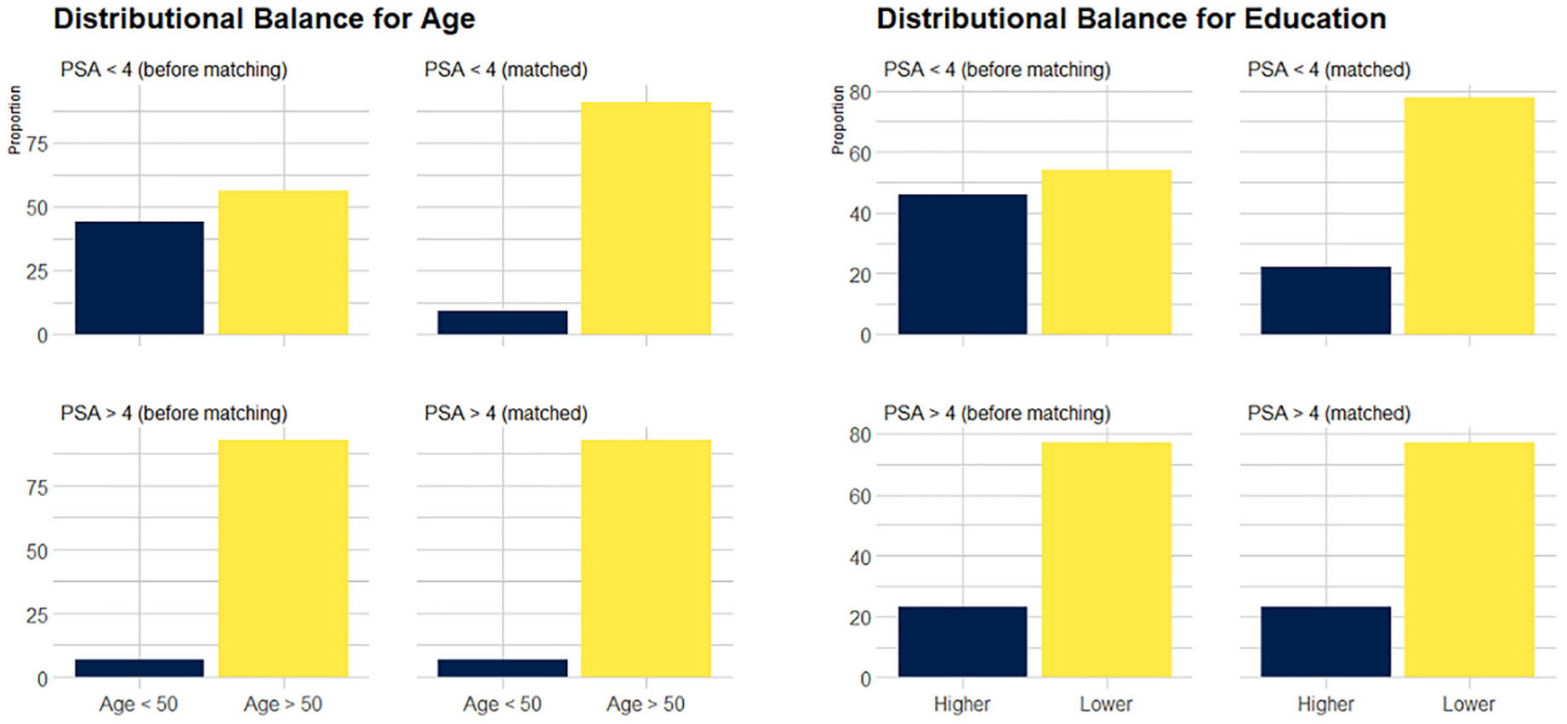
Visualization of distribution of covariates before and after matching.

The data obtained after applying PSM (1:12) are shown in **Table 3** and showed that balance was achieved. **Figure 1** shows the distribution of age and education before and after matching. The initial pool of participants with a PSA level of < 4 ng/mL, which included 3217 individuals in the full dataset, was meticulously narrowed to 1548 through the matching process. The multivariate logistic regression model showed that a low frequency of water intake (*P* <.01), WHR of >0.95 (*P* <.05), and a diagnosis of CMD (*P* <.05) were factors significantly associated with high serum PSA levels.

**Table 3:** Chi-squared test and logistic regression analysis after 1:12 propensity score matching.

Variables	Univariate			Multivariate		
	PSA<4 (N=1548) n (%)	PSA≥4 (N=129) n (%)	p	effect	SE	p
Age (years)			0.36			
>50	1402 (91)	120 (93)				
≤50	146 (9)	9 (7)				
Education level			0.77			
Low	1207 (78)	99 (77)				
High	341 (22)	30 (23)				
Vegetables (3 servings/day)			0.07			
Low	666 (43)	66 (51)				
High	881 (57)	63 (49)				
Water intake (2000 mL/day)			<0.01			0.002
Low	272 (28)	76 (37)		0.60	0.16	
High	699 (72)	129 (63)		–	–	
Waist-hip ratio (WHR)			<0.001			0.04
>0.95	587 (38)	64 (50)		0.38	0.19	
<0.95	945 (62)	64 (50)		–	–	
Cardiometabolic diseases			<0.01			0.04
Yes	725 (47)	76 (59)		0.38	0.19	
No	811 (53)	53 (41)		–	–	

PSA, prostate-specific antigen.

### 3.3 Analysis of PSA levels using genetic matching and multivariate logistic regression

**Figure 2:**
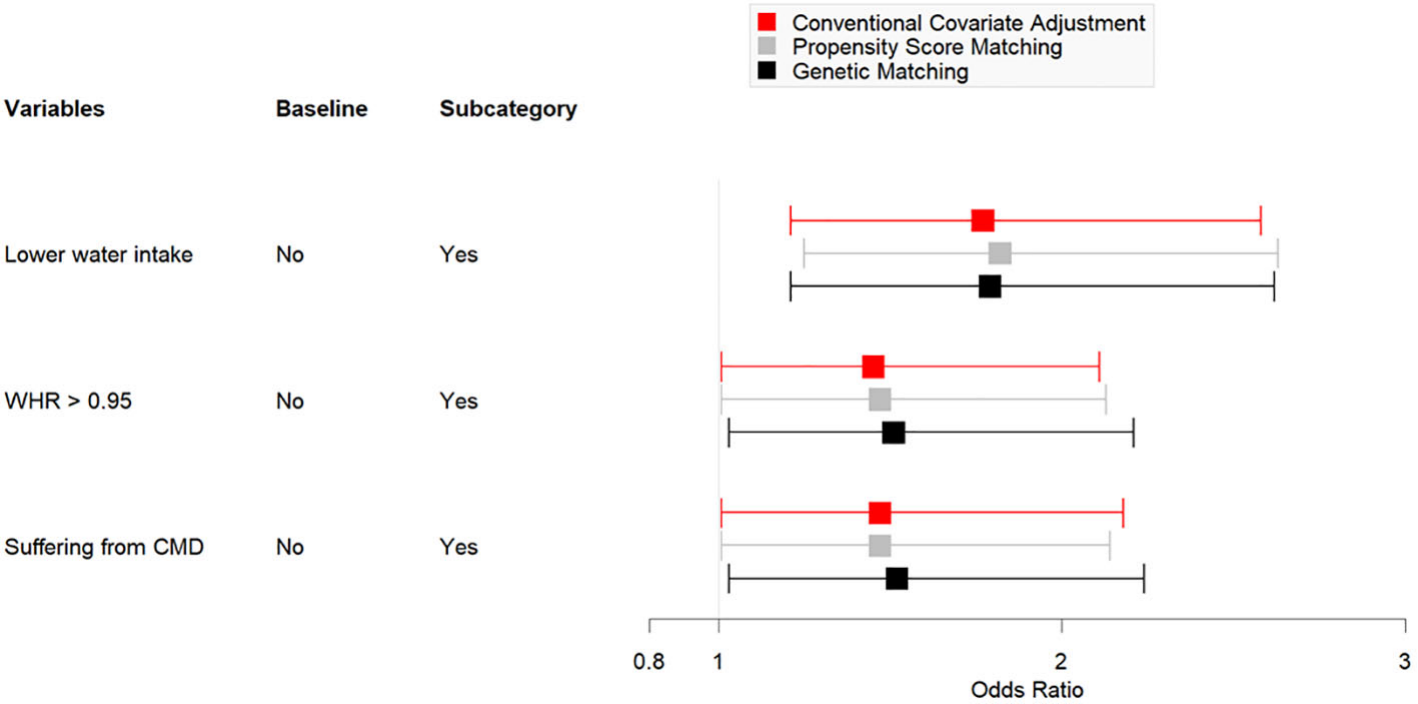
Comparison Chart of OR for Conventional Covariate Adjustment, PSM and GM Models.

**Table 4** shows the data obtained after employing GM (1:17, population size = 150). After matching for age and education, the number of men with PSA levels of < 4 ng/mL was 1233. Univariate analysis indicated homogeneity in the distribution of age and education level between the two groups (*P* >.05). The multivariate logistic regression model indicated that a low frequency of water intake (*P* <.01), WHR of > 0.95 (*P* <.05), and a diagnosis of CMD (*P* <.05) were significantly associated with high serum PSA levels. **Figure 2** presents a comparison chart of the odds ratios (OR) obtained through conventional covariate adjustment, PSM, and GM based on a multivariate logistic regression model. In the logistic regression analysis with conventional covariate adjustment, the 95% confidence intervals for low frequency of water intake, WHR of > 0.95, and presence of CMD all excluded the value 1.

**Table 4:** Chi-squared test and logistic regression analysis after 1:17 genetic matching.

	Univariate			Multivariate		
	PSA<4 (n=1233) n (%)	PSA≥4 (n=129) n (%)	p	effect	SE	p
Age (years)			0.08			
>50	1084 (88)	120 (93)				
≤50	149 (12)	9 ( 7)				
Education level			0.83			
Low	937 (76)	99 (77)				
High	296 (24)	30 (23)				
Vegetables (3 servings/day)			0.05			0.34
Low	520 (42)	66 (51)		0.18	0.19	
High	712 (58)	63 (49)		–	–	
Water intake (2000 mL/day)			0.003			0.003
Low	349 (29)	53 (41)		0.58	0.20	
High	875 (71)	76 (59)		–	–	
Waist-hip ratio (WHR)			0.005			0.03
>0.95	459 (38)	64 (50)		0.41	0.19	
<0.95	763 (62)	64 (50)		–	–	
Cardiometabolic diseases			0.004			0.03
Yes	557 (46)	76 (59)		0.42	0.20	
No	666 (54)	53 (41)		–	–	

PSA, prostate-specific antigen.

## 4 Discussion

This study provides valuable findings for the interpretation of serum PSA levels of ≥ 4 ng/mL and associated risk factors. It revealed two important findings. First, middle-aged men with high PSA levels had a high prevalence of unhealthy lifestyles, including adoption of a less healthy diet, low water intake, and physical inactivity. Second, high serum PSA levels in men were significantly associated with obesity and CMD after a sophisticated matching approach and covariate adjustment. These findings imply further alternative interpretations and health counseling for men when they misperceive PSA information from annual health checkups.

The consumption of adequate vegetables, fruits, and water is an essential component of a healthy diet (22, 29). Vegetables and fruits are rich in anti-inflammatory agents that can reduce oxidative stress (11, 30). Some studies have explored the relationship between fruit intake and serum PSA levels. For instance, research has shown a significant reduction in serum PSA concentrations among individuals consuming cranberry fruit over time and benefits for the prevention of cancer and urinary tract infections (13, 31, 32). Further, regular consumption of tomato products or red fruits/vegetables, which are rich in lycopene, is found to be beneficial against cancer-related symptoms and has been linked to a low risk of PCa (33–35). The present study indicated that many middle-aged men with high serum PSA levels did not consume adequate quantities of vegetables, fruits, and water. Previous studies have also indicated that insufficient water intake over a lengthy period may cause chronic dehydration, which is characterized by increased serum osmolality and affected by CMDs (29, 36). Chronic dehydration and elevated osmolality may exacerbate the effects of CMDs, which, in turn, could further influence PSA levels through mechanisms such as chronic inflammation and metabolic disturbances. This suggests that elevated PSA levels may not be driven by a single factor but rather by a combination of factors, including CMDs. Hence, education on adopting a healthy diet and adequate water intake for middle-aged men with high PSA levels is an important issue that should be addressed in further health promotion programs.

Some previous studies have demonstrated that regular exercise, including aerobic/resistance or high-intensity interval training, has a positive effect on patients with PCa and reduces serum PSA levels (37–39). For instance, meta-analyses have shown that exercise aids improve cancer-specific quality of life, cardiorespiratory capacity, resting fat oxidation, glucose levels, and body composition. Exercise has also been suggested to play a vital role as a complementary therapy for PCa and should be incorporated into treatment plans (37, 38). Moreover, some randomized controlled trials have provided robust evidence supporting high-intensity interval training as a crucial modifier of cardiorespiratory fitness and PSA levels in men with localized PCa under active surveillance. In previous studies, groups performing high-intensity exercise showed a decrease in PSA levels by 1.1 μg/mL, highlighting the potential of structured exercise regimens in influencing PSA levels. Furthermore, such exercise is beneficial for achieving higher lean mass levels and an improved nutritional status (23, 39, 40).

### 4.1 Limitations

This study was aimed at investigating the prevalence of and factors associated with high serum PSA levels in rural middle-aged men. While it has numerous strengths, including the use of community-based health screening and the incorporation of comprehensive physiological biomarkers from a standard central laboratory of a local hospital and health district, the results should be interpreted in light of several limitations. First, the study was conducted in only one county, which may have limited the generalizability of the findings. Second, this cross-sectional study could not determine causal relationships. Third, health-related behaviors, such as alcohol consumption and cigarette smoking, and CMDs were mostly self-reported, which might distort the study findings.

## 5 Conclusion

In this study, the prevalence of serum PSA levels of ≥ 4 ng/mL among rural men aged > 40 years was 3.9%. A low frequency of water intake, obesity, and a diagnosis of CMDs significantly contributed to high serum PSA levels. Therefore, in addition to being a tumor marker for PCa progression, PSA can be used in primary healthcare settings for the early detection and prevention of CMDs through individualized lifestyle modifications.

## Data Availability

The raw data supporting the conclusions of this article will be made available by the authors, without undue reservation.
